# Diagnosed Opioid Use Disorder Among Older Adults: Complex Comorbidity and Management Challenges

**DOI:** 10.1093/geroni/igaa057.172

**Published:** 2020-12-16

**Authors:** Peter Treitler, Stephen Crystal, Richard Hermida

**Affiliations:** Rutgers University, New Brunswick, New Jersey, United States

## Abstract

In the face of a widespread opioid epidemic and many policy changes affecting opioid access and management, it is important to understand the prevalence and characteristics of diagnosed opioid use disorder in older people and their implications for effective management of this high-risk population. We examined these issues in an ~40% random sample of Medicare beneficiaries with Part D coverage. In 2017, .8% of beneficiaries ages 65+ were diagnosed with OUD (opioid abuse or dependence diagnoses), an increase from .5% in 2015. The late-2015 transition from ICD-9 to ICD-10 may have contributed to this change, but the rate also increased post-ICD-10 by 9.1% from 2016-2017. The profile of individuals diagnosed with OUD reveals a population with complex comorbidity and multiple health challenges: 45% were diagnosed with major depression, 7% with alcohol disorders, 45% with anxiety, 8% with hepatitis C, 26% with cancer, 38% with COPD and 19% with pneumonia (risk factors for opioid overdose), 56% with diabetes and 27% with heart failure. 97% were diagnosed with pain conditions, 85% received opioid prescriptions, and 38% received benzodiazepine prescriptions. These patients represent complex and potentially competing challenges in concurrent management of pain, opioid use disorder, multi-substance use and opioid use disorder. Development of effective, integrated care models to simultaneously address these interrelated problems in this high-risk population should be informed by a closer focus on their multiple needs and monitoring of the adequacy of health system response.

